# Impact of COVID-19 on Urban Mobility and Parking Demand Distribution: A Global Review with Case Study in Melbourne, Australia

**DOI:** 10.3390/ijerph19137665

**Published:** 2022-06-23

**Authors:** Biruk G. Mesfin, Daniel(Jian) Sun, Bo Peng

**Affiliations:** 1 State Key Laboratory of Ocean Engineering, School of Naval Architecture, Ocean and Civil Engineering, Shanghai Jiao Tong University, No. 800 Dongchuan Road, Min-Hang District, Shanghai 200240, China; birukgmesfin@sjtu.edu.cn; 2 Smart City and Intelligent Transportation Interdisciplinary Center, College of Future Transportation, Chang’an University, Wei-Yang District, Xi’an 710021, China; jiansun@chd.edu.cn; 3 School of International and Public Affairs, Shanghai Jiao Tong University, No. 1954 Huashan Road, Xu-Hui District, Shanghai 200092, China

**Keywords:** COVID-19, urban mobility, parking demand, IoT parking sensors, explanatory data analysis, parking policies

## Abstract

The tremendous impact of the novel coronavirus (COVID-19) on societal, political, and economic rhythms has given rise to a significant overall shift from pre- to post-pandemic policies. Restrictions, stay-at-home regulations, and lockdowns have directly influenced day-to-day urban transportation flow. The rise of door-to-door services and the demand for visiting medical facilities, grocery stores, and restaurants has had a significant impact on urban transportation modal demand, further impacting zonal parking demand distribution. This study reviews the overall impacts of the pandemic on urban transportation with respect to a variety of policy changes in different cities. The parking demand shift was investigated by exploring the during- and post-COVID-19 parking policies of distinct metropolises. The detailed data related to Melbourne city parking, generated by the Internet of things (IoT), such as sensors and devices, are examined. Empirical data from 2019 (16 March to 26 May) and 2020 (16 March to 26 May) are explored in-depth using explanatory data analysis to demonstrate the demand and average parking duration shifts from district to district. The results show that the experimental zones of Docklands, Queensbery, Southbanks, Titles, and Princess Theatre areas have experienced a decrease in percentage change of vehicle presence of 29.2%, 36.3%, 37.7%, 23.7% and 40.9%, respectively. Furthermore, on-street level analysis of Princess Theatre zone, Lonsdale Street, Exhibition Street, Spring Street, and Little Bourke Street parking bays indicated a decrease in percentage change of vehicle presence of 38.7%, 56.4%, 12.6%, and 35.1%, respectively. In conclusion, future potential policymaking frameworks are discussed that could provide further guidance in stipulating epidemic prevention and control policies, particularly in relation to parking regulations during the pandemic.

## 1. Introduction

Since the primary case of COVID-19 was recorded in Wuhan, China, on 9 December 2019, there has been an alarming and rapid spread of the virus to every corner of the globe, highly catalyzed by the international passenger and cargo air transport network serving major cities in East Asia, the United States, and elsewhere [1]. Owing to the emergency, daily routines in cities throughout the world were chaotic during the first six months of 2020. The normal lives of people in many countries ceased as the pandemic crisis worsened. The human toll has been extensive due to the exponentially increasing number of cases and deaths around the world. Social distancing, stay-at-home regulations, and various other social restrictions have brought about a tremendous shift from the previously defined standards of human social interaction, such as greetings and hugging [2]. On the economic front, the endemic, epidemic, and then the pandemic has caused many businesses to delay or cease operations. Importing and exporting in most countries throughout the world have been highly disrupted due to the pandemic’s impact on the aviation industry [3,4,5], maritime industry [6], regional railway systems [7] and urban mobility [8]. In the manufacturing industry, sectors have been advised by the World Health Organization (WHO) to concentrate on producing medical supplies for fighting the pandemic, which has resulted in both collaborations and disputes regarding global transactions that involve medical supplies such as masks, gloves, and ventilators [9]. In regard to global politics, the pandemic’s impact has been dependent on nations’ health security strategy and emergency response capacity. The political impact has expanded due to significant global political decisions regarding emergency flight cancellations throughout the world. The status quo has impacted the aviation industry greatly, with 90% of passenger flights being cancelled [5]. In China, a number of airlines discontinued flights to and from the epicenter of the virus (Wuhan) and then across the entire country to avoid an escalation of the situation from epidemic to pandemic status. Moreover, following a loss of revenue, airlines throughout the world reacted to these changes by shifting from passenger to cargo flights, such as Ethiopian Airlines [10] and United Airlines [11].

As the pandemic impacted socioeconomic status, the political focus moved from the global to urban scale, with urban mobility being one of the most highly affected sectors. Modal travel shifts occurred, mainly on account of social distancing and contact-free policies. Urban mass transportation, such as car-sharing, ride-sharing, and ride-hailing, has been the most severely affected service types. Ride-sharing and taxi-hailing services, as well as urban electric commercial vehicles have been on the rise in recent years owing to AI-driven smart applications, and management and is favored due to a variety of factors, including minimized travel costs, traffic congestion, emissions and energy issues [12,13,14]. However, in the aftermath of the pandemic; for example, the ride-sharing market is predicted to have lost a quota share of 50% to 60% during 2020 [15], but this is anticipated to rise by 70% to 80% with new countermeasures, such as barriers between drivers and passengers to adhere to social distancing restrictions.

Acute social restrictions in several highly impacted cities with stay-at-home restrictions have resulted in the emergence of more door-to-door delivery services to satisfy the basic living requirements of those who cannot leave home. Moreover, panic buying and the fear of contamination during the crisis have encouraged many consumers to download smart applications from service providers such as grocery stores and restaurants. This app-based evolution has resulted in many grocery stores recording their highest ever daily revenues at the end of March 2020, with consumer spending up by 87.4% [16]. The nature of droplet transmission means that the most effective virus prevention is contact avoidance, which has led to mass-gathering restrictions for theaters, bars, sports clubs, and schools, while trips to pharmacies, grocery stores, and restaurants have increased. The shift of travel destination followed by parking demand shift forced the reshaping of parking facilities in order to satisfy the demand. Parking garages have been used as major emergency preparedness spaces and temporary medical centers. When considering vacant garages to micro-mobility maintenance disruptions and lay-offs, reacting to the demands of the public and ensuring the safety of core workers have been essential. To this end, different parking policies have been introduced, from the lessening of parking restrictions and penalties to fee adjustments.

The parking service providers highly affected by the pandemic include metropolises, academic institutions, and air terminals. Parking-related income is noteworthy for small cities, contributing to more than 30% of the yearly budget income, and there has been an estimated 90% income loss in this sector [17]. The question arises as to how countries will quantify and counter such socioeconomic and political disturbance. To investigate the impact of the pandemic on different sectors, a number of data-driven studies have been carried out globally. One of such interest is the MOBIS-COVID19 study [18], a joint project of ETH Zurich and the University of Basel, using mobile phone GPS tracking data from 3700 participants to examine the impact of COVID-19 in Switzerland. Conversely, on designing countermeasure frameworks, recent studies and approaches, such as avoid–shift–improve [ASI] [19] and the PASS approach [20], in which “P”, “A”, “S”, and “S” correspond to different stages of the pandemic comprising categorical targets of transportation users and service providers, as well as governments, have been utilized.

This study assesses the impact of COVID-19 via the visualized Melbourne dataset, released by the city data administration to promote understanding on the impact of COVID-19 on city mobility. The visualizing and comparing of on-street car parking sensor data for the two preferred periods, between 16 March and 26 May 2019 and 16 March and 26 May 2020 are carried out to quantify the impact of COVID-19 on different business districts and socioeconomic classes for better assessing the impact of COVID-19. The remaining part of the paper is structured as follows: Section 2 reviews the impact range of COVID-19 and analyzes the impact on urban mobility; Section 3 focuses on analyzing the impact of the pandemic on parking demand and revenue; Section 4 visualizes the parking demand variation during the pandemic via the case study approach, using Melbourne CBD parking data; Section 5 provides conclusions and future research recommendations.

## 2. Impact of COVID-19 on Urban Mobility

From world-wide expert survey results, Zhang et al. [21] presented significant impacts of COVID-19 on transport and logistics. Following the disturbance, many cities have enforced dynamic policy approaches to minimize the negative impact of the pandemic on the general public. The major approaches to reduce the risk of the pandemic include providing contactless mobility modes and creating open spaces, such as pedestrian space, cycle ways, and open street developments. A few summarized examples are provided in Table 1 to illustrate cities impacted by the pandemic and forced to shift mobility policy measures and responses.

## 3. Impact of COVID-19 on Parking Demand and Revenue

The parking system is a direct replica of the urban mobility disturbance by COVID-19. For instance, the parking demand to access essential destination’s parks increased during the pandemic [29], while on the other side, parking demand to public gathering places such as stadiums, theaters, malls, etc., dropped. Since the flare-up of COVID-19, the USD 131B parking industry has had difficulty, mostly in hotels and aviation vicinities, encountering a drop of 50–70% commuter parking movement and more than 95% accommodation revenue losses daily [30]. In addition, a reduction in income occurred from parking authorization with fewer parkers and from communities halting or unwinding authorization [31]. A small known reality is that municipalities’ budgets intensely depend on parking-related incomes. Some detailed illustrations are presented in Table 2, for indicating the dependence of cities on parking revenue [17].

### 3.1. COVID-19 Impact on On-Street Parking

To demonstrate the extent of the on-street parking affected by the pandemic, two studies are reviewed in this section: the smart parking system developers Smarking [17], which was carried out by a random sampling of 511 zones over the US (averaging 136 parking spaces), and IEM [32], which was studied on 650 installed PrestoSense parking sensors in different parts of Geneva, Switzerland.

**Figure 1:**
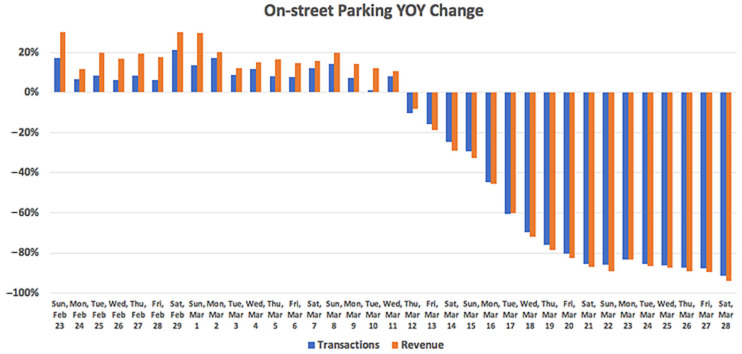
On-street parking year-over-year (YOY) change (from a random sample of 511 zones/blocks across the US).

The Smarking [17] survey showed a decrease in parking on the street at 80–90% since the US national emergency statement issued on Friday, 13 March 2020. Before and after the COVID-19 outbreak, the annual dynamics of parking activities have changed a lot: a year-over-year increase in 10%, to 80–90% decrease, compared to the same period last year (See Figure 1). Similarly, the peak daily occupancy of on-street parking spaces surveyed across the country shows that the pattern before and after Friday, 13 March has changed significantly.

Conversely, IEM [32] studied data from 650 PrestoSense parking monitoring sensors installed in different regions of Geneva, Switzerland, to illustrate how the parking patterns on the streets have changed in areas where semi-confinement was announced. This study focused on parking demand (in terms of occupancy and turnover) fluctuation before and after the emergency declaration date of 16 March 2020. The state of emergency was declared between 16 March and 11 May 2020, in which the semi-confinement period spanned from 16 March to April, and the progressive de-confinement period spanned from April to 11 May 2020.

During the semi-confinement period, parking restrictions such as vehicle parking control were suspended in Geneva. To analyze the policy shift impact, the PrestoSense vehicle detection sensors were installed in on-street parking bays of residential areas, offices and cultural areas to detect vehicle occupancy and the parking duration of a parked vehicle.

**Figure 2:**
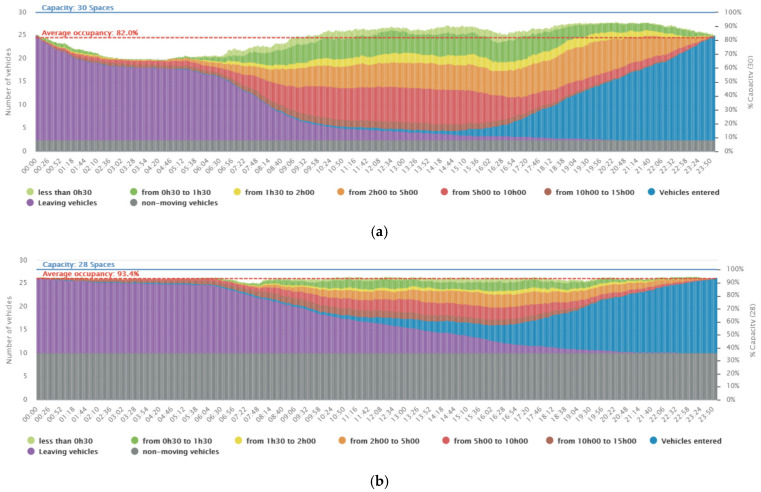
Average occupancy of the selected residential area (Rue des Pâquis, Geneve): (**a**) before the confinement period (between 5 January and 5 February 2020), and (**b**) during the confinement period (between 1 and 30 April 2020).

The on-street parking demand comparison assessment was performed between residential areas versus offices and cultural areas. In residential complexes, before the semi-confinement period, the parking usage in residential areas showed an average occupancy level of 82% (see Figure 2a) with an average car turnover rate of 7.2 per day; here, the turnover rate is defined as the average number of vehicles using the same parking space at a certain time gap. In the same area during the semi-confinement period, an increase in the occupancy level to 93.4% (see Figure 2b) and a decrease in car turnover rates to 3.2 cars per day was clearly observed.

**Figure 3:**
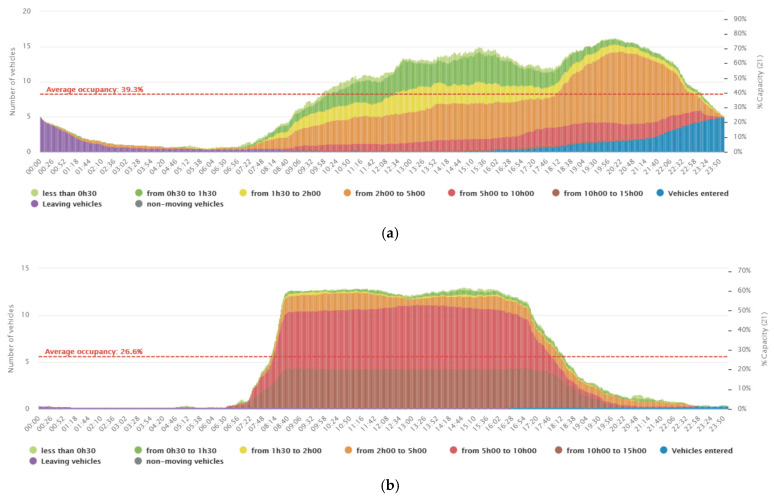
Average occupancy of the selected Office and Cultural site (Bd. Du théâtre, Geneve): (**a**) before the confinement period (between 5 January and 5 February 2020), and (**b**) during the confinement period (between 1 and 30 April 2020).

Meanwhile, in office and cultural sites, before the semi-confinement period, an average occupancy level of 39.3% (see Figure 3a), and car turnover rate of 5.7 cars per parking space per day was observed, whereas in the same area during the semi-confinement period, a substantial drop in occupancy levels to 26.6% (see Figure 3b) and a decrease in car turnover rates to 1.7 per parking space per day was marked.

### 3.2. COVID-19 Impacts on Off-Street Parking

**Figure 4:**
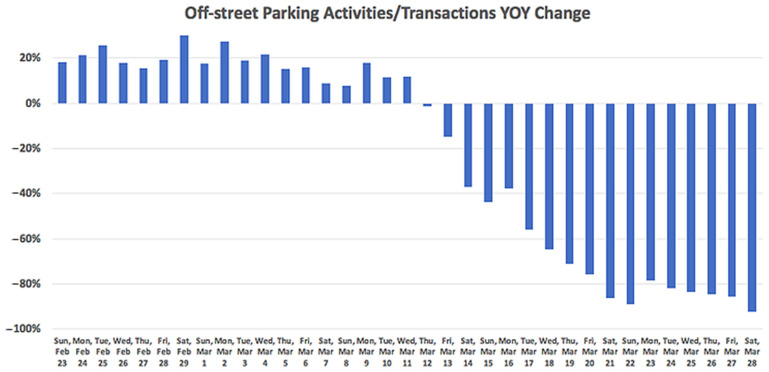
Off-street parking activities YOY change (from a random sample of 511 zones/blocks across the USA).

A study [17] under this topic was reviewed to show the impact of COVID-19 on off-street parking garages. During the investigation, a collection of 935 off-street parking locales, consisting of both private and city-owned parking garages was studied. The results show a comparative drift from on-street parking prior to the COVID-19 emergency: 10% YOY increment (see Figure 4). Then, after 13 March 2020, the parking demand dropped dramatically by about 90%.

## 4. Case Study: Melbourne City, Australia

To investigate the impact of COVID-19 on parking demand distribution on different types of urban areas and streets, this study used the parking data from Melbourne CBD parking bays, Australia, visualized to illustrate how the parking demand on different central districts was shifted by the pandemic. The in-ground parking sensors recorded vehicle arrival and departure times (from 1 January to 1 May 2020), which were released upon customer request to grasp the strike of COVID-19 on city movement. On-street car parking sensor data for 2019 [33] and from January–May, 2020 [34] were obtained to visualize the impact variation of COVID-19 on parked vehicle volume and average parking duration. Visualizing these parking sensors data will help to illustrate how the parking demand in terms of parking volume and average parking duration on different central districts is shifted by the pandemic during the state of emergency. The state of emergency was declared by the Minister for Health on 16 March 2020, under section 198 (1) of the Public Health and Wellbeing Act 2008 (Act) throughout the state of Victoria due to the serious threat to public health in Victoria due to the novel coronavirus 2019 [35].

On this case study, Melbourne city areas and streets were selected and analyzed to assess how the city areas and streets are impacted negatively with respect to parking demand after the first stage of the state of emergency dated from 16 March 2020. The assessment area selection criteria included the high shift in parking volume after the state of emergency versus the rest of the areas listed on the dataset, and the authors think the picked areas for analysis, named as Docklands, Queensbery, South banks, Titles, and Princess Theatre will illustrate the image for the high impact of COVID-19 on parking demand distribution. Further, at street-level impact analysis, streets of Princess Theatre were evaluated with respect to parking volume and parking duration indicators. The streets of Princess Theatre were chosen for analysis since this area shows a higher shift in parking demand out of the five selected areas.

The impact assessment and comparison were conducted between two periods, 16 March–26 May 2020 versus 16 March–26 May 2019. The parking demand indicators are parking volume, in terms of vehicles present, and average parking duration. The sensors read whether there is a parked vehicle or not, in which the data are given by (false, true) or (0, 1). The sum of the values “true” or “1” gives the total number of vehicles present or parked at a certain location during a certain period. The average parking duration was obtained by dividing the parking load (vehicle-minutes) by the total number of vehicles present during the entire duration of the assessment (Equation (1)). It can also be calculated as the sum of parking duration of all vehicles divided by the total number of vehicles parked during the survey period.

(1) $$\mathrm{Average}\;\mathrm{Parking}\;\mathrm{duration}\;\left(\mathrm{minutes}\right)=\frac{\mathrm{Parking}\;\mathrm{load}\;\left(\mathrm{veh}.\;\mathrm{minutes}\right)}{\mathrm{Parking}\;\mathrm{volume}\;\left(\mathrm{veh}.\right)}$$

**Figure 5:**
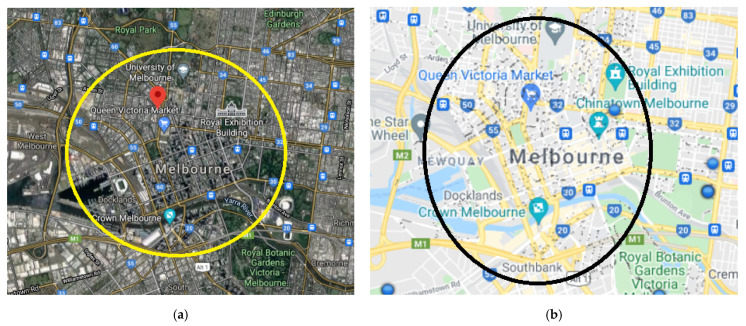
Study area: (**a**) Melbourne CBD, VIC, Australia; (**b**) on-street parking bays.

The authors believe that the study area (Figure 5) is planned to present the interior CBD of Melbourne. The black dots in Figure 5b show the on-street parking bays in the selected experimental areas of Melbourne streets [36].

### 4.1. Areal Based IMPACT Analysis

**Figure 6:**
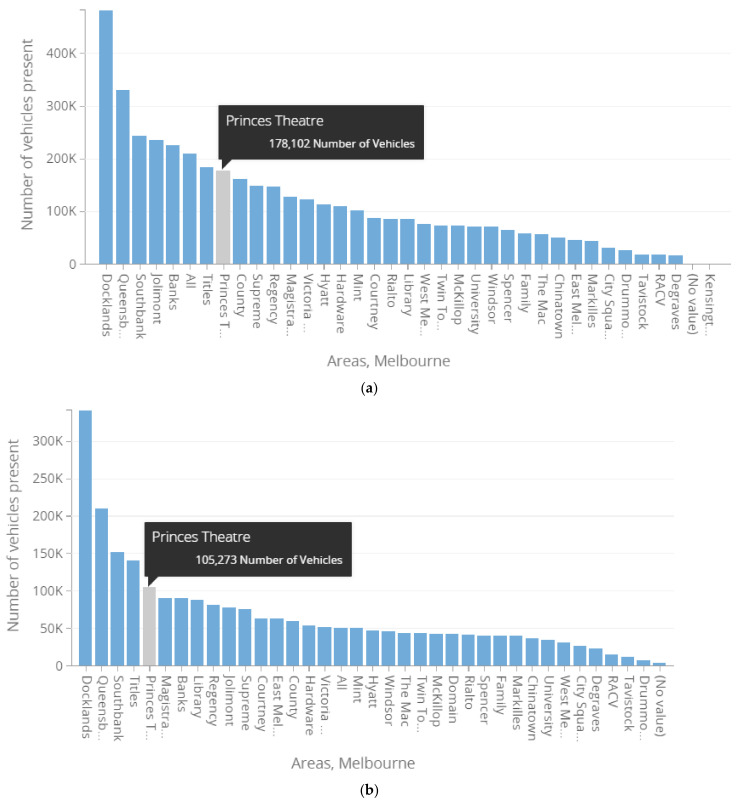
Bar graph representation of Princess Theatre’s area parking volume in terms of vehicles present between two periods: (**a**) 16 March to 26 May 2019 versus (**b**) 16 March to 26 May 2020.

**Parking volume comparison:** After the state of emergency, the vehicles present in on-street parking bays showed a significant decrease. For almost two months from 16 March to 26 May 2020, Docklands, Queensbery, Southbanks, Titles, and Princess Theatre areas showed a decrease in percentage change of vehicles present of 29.2%, 36.3%, 37.7%, 23.7%, and 40.9%, respectively, compared to the same period of the previous year from 16 March to 26 May 2019. Princess Theatre appeared to have the highest fall in parking volume percentage change from the picked experimental areas. As presented in Table 3 and Figure 6, the on-street parking spot demand decreased relatively within these periods, mainly due to the state of emergency restrictions, including the advice to stay home.

**Figure 7:**
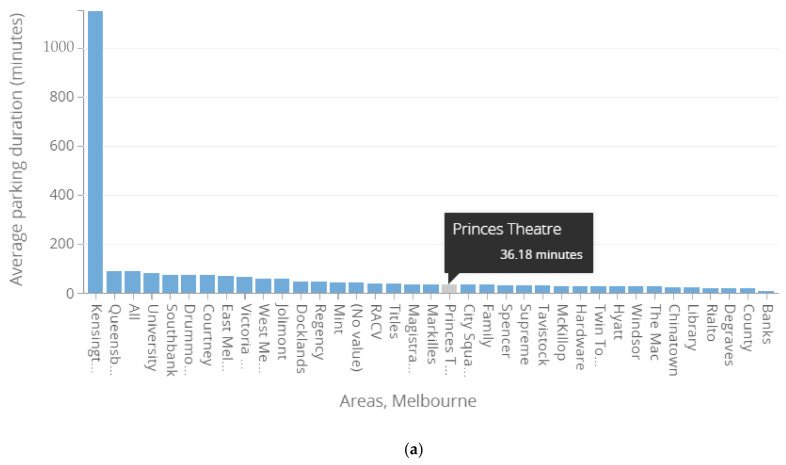

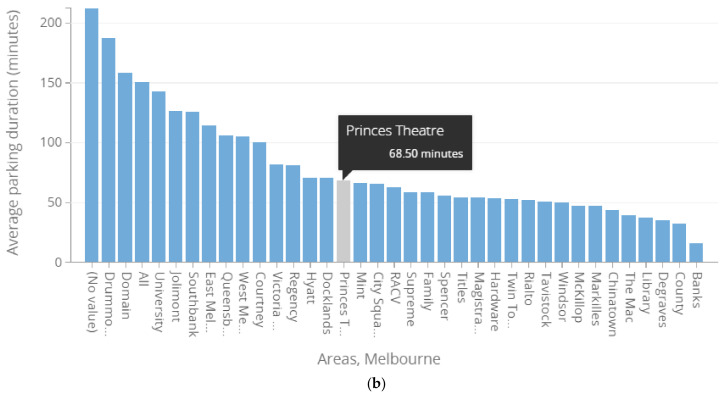
Bar graph representation, referring to the Princess Theatre area average parking duration between two periods: (**a**) 16 March to 26 May 2019 versus (**b**) 16 March to 26 May 2020.

**Average parking duration comparison:** After the state of emergency from 16 March to 26 May 2020, the average parking duration of on-street parking bays showed a significant increase. As shown in Table 4 and Figure 7, Docklands, Queensbery, Southbanks, Titles, and Princess Theatre areas showed an increase in percentage change of average parking duration of 45.3%, 17.9%, 64.5%, 38.2% and 89.3%, respectively, compared to the same period of the previous year from 16 March to 26 May 2019. Princess Theatre appeared to have the highest increase in average parking duration percentage change from the picked experimental areas. Referring to Table 3 and Table 4, Princess Theatre is the area that experienced the highest drop in vehicles present, with −40.9%, and the highest increase in percentage change of average parking duration, with +89.3%. This is mainly due to the socio-economic nature of the Princess Theatre area, which is famous for being an entertainment and gathering site [37]. The reason for the high shift in parking demand of this kind of areas was the state of emergency and restriction on gathering activities. Technically, the average parking duration (in min.) is defined as the ratio of total vehicle hours to the number of vehicles parked, i.e., parking load in Veh. min over parking presence (in Veh.). Therefore, mathematically, as a vehicle parking presence decreases with a wider gap than the parking load, the average parking duration may increase in a significant range.

### 4.2. Street Level Impact Analysis

For this part of the impact assessment, the streets circling Princess Theatre area (Lonsdale Street, Exhibition Street, Spring Street, and Little Bourke Street) were selected for parking demand visualization.

**Parking volume comparison:** After the state of emergency, the vehicles present in on-street parking bays showed a significant decrease. For almost two months from 16 March to 26 May 2020, Lonsdale Street, Exhibition Street, Spring Street, and Little Bourke Street parking bays showed a decrease in percentage change of vehicles present of 38.7%, 56.4%, 12.6%, and 35.1%, respectively, compared to the same period of the previous year from 16 March to 26 May 2019.

Exhibition Street appeared to have the highest fall in parking volume percentage change from the streets around Princess Theatre. As presented in Table 5, Exhibition Street, with a 56.4% drop in parking volume, lost the proportion of vehicles present within the area, from 27% to 20%. This is mainly because Exhibition Street is famous for being home to many heritage buildings, such as the Victorian Heritage Register [38], which are classified as one of Melbourne’s visiting sites, and which was highly impacted by the COVID-19 state of emergency measures.

**Average parking duration comparison:** After the state of emergency from 16 March to 26 May 2020, the average parking duration of the selected streets’ on-street parking bays showed a significant increase. As shown in Table 6, Lonsdale, Exhibition, Spring, and Little Bourke streets showed an increase in percentage change of average parking duration of 84%, 103.6%, 153.4%, and 58.3%, respectively, compared to the same period of the previous year from 16 March to 26 May 2019.

Spring Street appeared to have the highest increment in average parking duration percentage change from the streets around Princess Theatre, and Little Bourke Street with a 58.3% increment in average parking duration lost the proportion of average parking duration in the area, from 31% to 25%. This is mainly because Little Bourke Street is famous for its department stores and many boutiques [39], in which most of them were closed in relation to the COVID-19 state of emergency measures, having a direct impact on the interest of on-street parking from visitors.

## 5. Conclusions

As presented in the previous section, the parking volume and average parking duration were highly impacted due to the COVID-19 state of emergency in Melbourne city. Areal/zonal-based impact analysis, from 16 March to 26 May 2020, in Docklands, Queensbery, Southbanks, Titles, and Princess Theatre areas showed a decrease in percentage change of vehicles present of 29.2%, 36.3%, 37.7%, 23.7%, and 40.9%, respectively, compared to the same period of the previous year from 16 March to 26 May 2019. Then, considering the average parking duration as a parameter, Docklands, Queensbery, Southbanks, Titles, and Princess Theatre areas showed an increase in percentage change of average parking duration of 45.3%, 17.9%, 64.5%, 38.2%, and 89.3%, respectively, within same period.

Furthermore, the street-level impact analysis of the Princess Theatre area, Lonsdale Street, Exhibition Street, Spring Street, and Little Bourke Street parking bays showed a decrease in percentage change of vehicles present of 38.7%, 56.4%, 12.6%, and 35.1%, respectively. With the same parametrical comparisons as the zonal analysis, Lonsdale, Exhibition, Spring, and Little Bourke streets showed an increase in percentage change of average parking duration of 84%, 103.6%, 153.4%, and 58.3%, respectively. The variation in the impact of the pandemic might come from a different basis. The social, infrastructural, and institutional nature of the urban areas and streets might have been impacted by the pandemic regarding parking demand sensitivity.

Quantifying the impact throughout the urban area might be useful for implementing different types of parking policies and interventions and to further help maximizing the performance of COVID-19 urban mobility measures. The massive declines on intercity transportation brought the direct drop in parking demand. Cities are scrambling to recover not only the lost parking revenues, but also the significant reduction in fees and fines. As parking is one of the significant incomes of most municipalities, the decline in income has resulted in tremendous challenges to support their health care system.

Analyzing the mitigation measures and preparing for the coming “new normal” [40,41] may assist in understanding the opportunities behind the risks regarding parking industries. When dealing with policies and measures during the pandemic, it is critical to understand the interrelationship between jobs and food access, housing, public health, and mobility. The pandemic is an opportunity for private and public parking transit operators to advance mobility and parking needs and to strengthen economic resilience, particularly for the most vulnerable populations with equity and accessibility concepts.

The parking industry response implementation from COVID-19 might include three basic creative approaches of adaptiveness, pivoting needs, and replacing the lost revenue approach [30]. Parking lots have been adapted to be used as COVID-19 screening and as real-time tracking platforms that support the city’s requirements with all the parking KPIs and the most up-to-date information accessible for the governmental task forces. The common topics on pivoting demands are that the industry should repurpose, be agile, and be adaptable during this time. A few ways for pivoting the needs in times of instability to preserve the quality of the parking and mobility programs can be framed as operational and financial sections. In operational prioritizing: moving all customer support to a virtual environment, transitioning enforcement staff to assist with healthcare operations, and renegotiating travel contracts to lower rates and move transport drivers to on-demand benefits. In financial prioritizing: plan and finance the year into smaller fiscal budgets rather than completing the full budget process (due to the flexible nature of the pandemic impact) and arrange to prioritize socio-economic zones based on needs targeting equity, accessibility, and vulnerability. In addition, a fast recovery plan to support the potential revenue loss has to be developed and implemented as early as possible. Financial models are required to understand the revenue impacts on the ground, with varying possibilities on the expected saturation curve and with further extension for the coming years. Understanding the pandemic projection, the estimated saturation line, and the nation’s health institutions’ capacity helps to design an acceptable and successful financial recovery plan.

Planners and specialists witness new opportunities to sustain their projects, which mainly push for smart working and reduce unnecessary labor intensity. The COVID-19 pandemic may require to examine the operations holistically and to be smarter about our future programs. Some potential positive outcomes are becoming more virtual and customer friendly, expanding and exploring new technologies, and aligning parking pricing models with the true cost of our parking and mobility programs. Following this, the COVID-19 impact on parking policy change in relation with the demand shift of commercial ridesharing vehicles can be modeled by discrete decision models, which introduce a behavioral framework regarding a commercial vehicle parking choice [42]. With additional considerations regarding the expected future COVID-19 factors, the model may be used for capturing pick and delivery vehicles regarding parking policy sensitivity.

Future CITY-20′s studies of post-COVID-19 parking and urban space trends are divided into three categories: **Smart Parking and Curb Management**, **Artificial Intelligence and Data Driven Technology**, and **Autonomous Vehicles (AVs)**. Smart parking management and enforcement ethos can cure almost all parking problems during the emergency period of the pandemic. Real-time IoT parking solutions and management are expected to be used in future applications to optimize parking operation and management [43,44]. COVID-19 has also expedited the improvement in artificial intelligence in numerous diverse aspects representing a lessening of human contact or nearness, which furthermore may be regarded as a catalyst for the advancement of open source information and data. For instance, recent publications such as [45,46,47] have mined and structured big data to forecast and predict this and other illnesses. The rise of AVs may be placed in public transportation services, which may serve as an advanced arrangement to defend the transit workforce.

## Figures and Tables

**Table 1:** Summary for mobility policy measures and responses.

City, Country	Measures and Responses
Paris, France [22]	The COVID-19 pandemic accelerated/prioritized the implementation of Paris Mayor Anne Hidalgo’s 2024 vision for 650 km of cycle.
Milan, Italy [23]	35 km of streets were repurposed and made open for active modes of transportation, and a 30 km/h speed limit was introduced.
Bogota, Colombia [24]	The Colombian capital has opened 76 km of temporary bicycle lanes, and 22 km of the new lanes were opened on 17 March 2020 by repurposing vehicle lanes.
Budapest, Hungary [25]	Bike lanes were added at the edges of multi-lane streets.
Brussels, Belgium [26]	The government has created 40 km of new cycle paths and turned the city center into a 20 km long walkable area.
Auckland, New Zealand [27]	The first authorities to fund pop-up bike lanes, widened sidewalks and related infrastructures during lockdown.
Helsinki and Espoo, Finland [28]	The COVID-19 outbreak forced the city to open the bike season earlier than the previous year with a total of 351 bike stations (242 in Helsinki and 109 in Espoo).

**Table 2:** Municipalities budget from parking-related revenues (in United States Dollars, 2019–2020 fiscal year).

Cities	Total Parking Related Revenue	Parking Tax	Fees, Fines, and Citations
San Francisco	USD 439.7M	USD 85.5M	USD 354.2M
New York	USD 1.1B+	USD 300M	USD 785M
Chicago	USD 299.7M	USD 134M	USD 25.1M

**Table 3:** Area level comparisons of parking volume in terms of number of vehicles present between two periods (16 March to 26 May 2019 versus 16 March to 26 May 2020).

Area	Number of Vehicles Present		Percentage Change
	16 March–26 May 2019	16 March–26 May 2020	
Docklands	482,426	341,538	−29.2%
Queensbery	330,106	210,257	−36.3%
Southbanks	243,897	151,845	−37.7%
Titles	184,969	141,161	−23.7%
Princess Theatre	178,102	105,273	−40.9%

**Table 4:** Area level comparisons of the average parking duration change in minutes between periods (16 March to 26 May 2019 versus 16 March to 26 May 2020).

Area	Average Parking Duration (min)		Percentage Change
	16 March–26 May 2019	16 March–26 May 2020	
Docklands	48.65	70.71	45.3%
Queensbery	90.15	106.29	17.9%
Southbanks	76.51	125.88	64.5%
Titles	39.51	54.61	38.2%
Princess Theatre	36.18	68.5	89.3%

**Table 5:** Street level comparisons of the parking volume in terms of the change in number of vehicles present between two periods (16 March to 26 May 2019 versus 16 March to 26 May 2020).

Princess Theatre Streets	Number of Vehicles Present		Percentage Change
	16 March–26 May 2019	16 March–26 May 2020	
Lonsdale street	112,863	69,166	−38.7%
Exhibition street	47,299	20,630	−56.4%
Spring street	8040	9054	−12.6%
Little Bourke street	9900	6423	−3.51%

**Table 6:** Street level comparisons of the average parking duration change, in minutes, between two periods (16 March to 26 May 2019 versus 16 March to 26 May 2020).

Princess Theatre Streets	Average Parking Duration (min)		Percentage Change
	16 March–26 May 2019	16 March–26 May 2020	
Lonsdale Street	35.42	65.18	84%
Exhibition Street	37.19	75.71	103.6%
Spring Street	29.17	73.93	153.4%
Little Bourke Street	45.45	71.96	58.3%

## Abbreviations

AI	artificial intelligence
APP	smart mobile/computer/Android/TV applications
AV	autonomous vehicles
CBD	Central Business District
COVID-19	Coronavirus disease 2019
EIT	European Institute of Innovation and Technology
IATA	International Air Transport Association
ICAO	International Civil Aviation Organization
IEM	Ingenierie Electronique et Monetique
IoT	Internet of Things
MOBIS	Mobility Behaviour In Switzerland
TUMI	Transformative Urban Mobility Initiative
WHO	World Health Organization
YOY	year-over-year
